# Lysine Malonylation and Its Links to Metabolism and Diseases

**DOI:** 10.14336/AD.2022.0711

**Published:** 2023-02-01

**Authors:** Lu Zou, Yanyan Yang, Zhibin Wang, Xiuxiu Fu, Xiangqin He, Jiayi Song, Tianxiang Li, Huibo Ma, Tao Yu

**Affiliations:** ^1^Institute for Translational Medicine, The Affiliated Hospital of Qingdao University, Qingdao, China.; ^2^Department of Immunology, Basic Medicine School, Qingdao University, Qingdao, China.; ^3^Department of Cardiac Ultrasound, The Affiliated Hospital of Qingdao University, Qingdao, China.; ^4^Department of Vascular Surgery, The Affiliated Hospital of Qingdao University, Qingdao, China.

**Keywords:** malonylation, metabolism, angiogenesis, fatty acids oxidation, cardiomyopathy

## Abstract

Malonylation is a recently identified post-translational modification with malonyl-coenzyme A as the donor. It conserved both in prokaryotes and eukaryotes. Recent advances in the identification and quantification of lysine malonylation by bioinformatic analysis have improved our understanding of its role in the regulation of protein activity, interaction, and localization and have elucidated its involvement in many biological processes. Malonylation has been linked to diverse physiological processes, including metabolic disorders, inflammation, and immune regulation. This review discusses malonylation in theory, describes the underlying mechanism, and summarizes the recent progress in malonylation research. The latest findings point to novel functions of malonylation and highlight the mechanisms by which malonylation regulates a variety of cellular processes. Our review also marks the association between lysine malonylation, the enzymes involved, and various diseases, and discusses promising diagnostic and therapeutic biomolecular targets for future clinical applications.

## 1. Introduction

Post-translational modifications (PTMs) play an important regulatory role in most aspects of cell biology, including pathological and physiological processes. They exert this role by regulating protein function through covalent chemical modifications of amino acid side chains or protein ends or by modifying the structure and function of amino acid side chains [1-3]. PTMs can change the physicochemical properties and conformations of proteins, thereby altering their function and binding ability. Therefore, although expression levels of proteins may not change, their function changes based on the state of PTM and can be amplified. PTMs affect protein function in diverse manners:(1) the same protein can perform multiple functions, even with a single type of modification; (2) the same PTM of a protein may result in different functions if it occurs on different amino acids; and (3) the same protein may have different modifi-cations, with more complex functions and involved biological processes [4]. PTMs participate in almost all aspects of biology [5], and their aberrant states are implicated in many diseases, including metabolic diseases, cancer, growth and developmental disorders, and cardiovascular diseases [6-9]. Thus, PTM research is crucial to the elucidation of epigenetic mechanisms related to the occurrence and progression of various diseases [10].

Lysine is a basic essential amino acid whose chemical name is 2,6-diaminohexanoic acid. Protein modifications usually occur on unstable amino acid side chains, such as that of lysine. Lysine has been observed to occur with high frequency in the predicted modification sites of proteins. Advances in experimental techniques have led to the identification of various types of lysine PTMs. More than 20 lysine PTMs have been characterized and annotations deposited in public databases, such as the Protein Lysine Modification Database [11] and the Phosphorylation Sites Plus [12]. The variety in lysine PTMs [13] and the crosstalk between lysine PTMs and other PTMs demonstrate its importance and highlight its indispensable functions in different biological processes [14].

Lysine malonylation is an evolutionarily conserved acyl modification, that was discovered in 2011 in mammals and *Escherichia coli*, using high-throughput proteomic analysis [15]. The modification requires malonyl-coenzyme A (CoA) as a substrate, which is added to the lysine residue, and regulates protein structure and function by interfering with electrostatic interactions (from +1 to -1). Lysine malonylation has important roles in various metabolic processes and stress responses [16-19]. Malonylation modifications are closely related with metabolic diseases, such as inflammation and type 2 diabetes, and angiogenesis-associated diseases. Angio-genesis is the formation of new blood vessels from pre-existing ones through sprouting. Growing evidence suggests that epigenetic modifications, such as DNA methylation [20], histone acetylation [21], and histone lactylation [22], are implicated in angiogenesis and vascular defects [23]. In addition, lysine malonylation is abundant in mitochondrial proteins that regulate metabolic pathways, including glycolysis, fatty acid oxidation, and inflammation, particularly in endothelial cells (ECs) and macrophages [24].

Emerging discoveries on malonylation have allowed researchers to shift focus from malonyl-CoA to malonylation and the signaling pathways involved in the regulation of multiple diseases. Available literature has demonstrated the potential clinical value of lysine malonylation. In this review, we discuss the regulatory role of malonylation, the underlying mechanisms, and describe its close association with various diseases.

## 2. Malonylation-related enzymes

**Figure 1.**
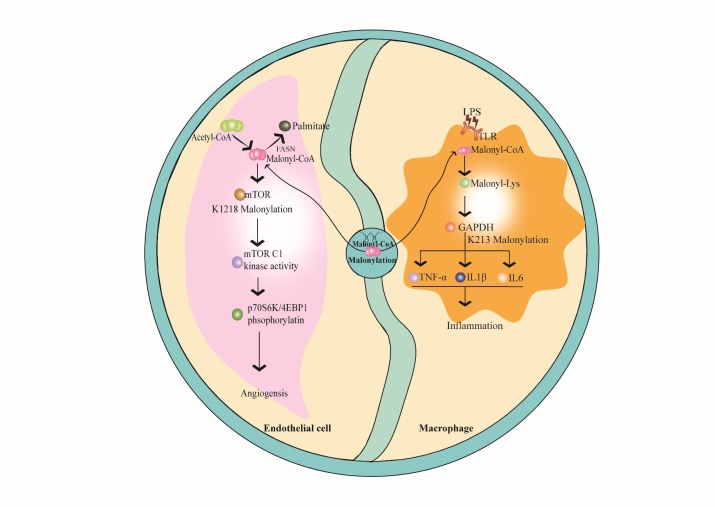
Production of malonyl-CoA and its regulation in malonylation. Glucose, amino acid and citrate metabolism all lead to the production of malonyl-CoA. The concentration of malonyl-CoA can be balanced by the action of ACC1 and MCD. Mitochondrial malonyl-CoA is produced by the malonyl coenzyme A synthase ACSF3. Sirt5 acts as a demalonylase, catalyzing a deacylation (demalonylation) reaction that consumes NAD^+^ as a co-substrate and generates NAM, 2'-O-malonyl-ADP-ribose, and the deacylated product. Malonyl-CoA inhibits the acyltransferase activity of CPT1. Acetyl-CoA, Acetyl Coenzyme A; ACC1, acetyl-CoA carboxylase 1; MCD, Malonyl-CoA decarboxylase; Malonyl-CoA, Malonyl-Coenzyme A; Sirt5, Sirtuin 5; NAM, Nicotinamide; ACSF3, Acyl-CoA Synthetase Family Member 3; CPT1, carnitine palmitoyltransferase 1.

PTMs expand the functional proteome by regulating the functions and structures of proteins. The enzymes regulating PTMs, therefore, act as central regulators of cell signaling [25]. PTM enzymes are substrate-specific, a property that determines their functions in regular physiological processes as well as in disease conditions. Reader protein and enzyme networks typically control gene transcription through dynamic removal, addition, and recognition of PTMs on histone tails and other regulatory proteins [26, 27]. Reversible Nε-lysine acetylation/deacetylation is regulated by histone deacetylases (HDACs) and protein acetyltransferases (HATs). HDACs deacetylate their targets and regulate expression levels of the proteins involved in cell cycle and apoptosis. These proteins are often implicated in tumor growth, invasion, and drug resistance [28]. On the other hand, fewer studies have focused on the significance of the lysine malonylation protein networks in these processes. In this review, we discuss information from the most pertinent of these studies (Table 1, Fig. 1).

**Table1:** Enzymes involved in malonylation.

Enzyme	Localization	Function	Disease	References
Malonyl-CoA	Cytoplasmic	A donor of malonylation, Generate acetyl-CoA	Vascular defects, Osteoarthritis, Malonic aciduria, Inflammation	[15, 32, 34, 35, 117]
Malonyl-CoA decarboxylase	Cytoplasmic	Degradation of Malonyl-CoA, Demalonylation	Malonic aciduria	[37, 38]
Sirtuin 5	Cytoplasmic	Demalonylation *in vitro* and *in vivo*, Regulate glycolysis	Cardiovascular disease	[15, 18, 29-31]

Sirtuin 5 (SIRT5) is a mitochondrial desuccinylase, demalonylase, and lysine deacylase [29]. As a global regulator of lysine malonylation, SIRT5 has been reported to preferentially remove negatively charged carboxyl and acyl moieties. It acts as a catalyst in the removal of malonyl groups from the lysine side chains of protein substrates [30], resulting in reversible protein malonylation. This reaction is nicotinamide adenine dinucleotide (NAD^+^)-dependent; it consumes NAD^+^ as a co-substrate and produces the Sirtuin feedback inhibitor nicotinamide, 2'-O-malonyl-ADP-ribose and a deacylated substrate [15, 31].

A total of 430 proteins associated with 1,137 unique malonyl-lysine sites have been identified in mouse livers, assessed using label-free quantification and affinity enrichment [18]. In the absence of SIRT5, 10 of these sites were observed to be highly malonylated (≥1.5-fold change), indicating that 28% of malonylated proteins are SIRT5 targets, and 16% of malonyl-lysine sites are regulated by SIRT5. Fatty acid β-oxidation, glycolysis, gluconeogenesis, and the urea cycle are significant components of cellular metabolic networks and are among the most dynamically regulated pathways. SIRT5 regulates the activity of the glycolytic enzyme GAPDH, and glycolytic flux is inhibited in primary hepatocytes in the absence of SIRT5 [18]. These findings demonstrate the latent regulatory mechanisms of malonylation, provide insights into malonylation regulation by SIRT5, and present a general analysis of lysine malonyl groups *in vivo*.

Malonyl-CoA can function as a universal donor in malonylation reactions, possibly through non-enzymatic mechanisms or specific malonyltransferase activity, and is an important intermediate in the cellular metabolic pathway. It inhibits fatty acid oxidation and participates in a variety of pathological and physiological reactions. It can form an active acyl-CoA species with a carbonyl group via a thioester bond to the carbon chain [32]. Malonyl-CoA synthesis is suggested to occur through three major pathways: catalysis of acetyl-CoA carboxylation by (1) acetyl-CoA carboxylase (ACC), (2) propyl-CoA carboxylase, and (3) β-oxidation of odd-chain-length dicarboxylic acids [33]. Two isoenzymes of ACC have been identified in mammals [34] with different physiological functions, ACC1 and ACC2. ACC1 is a cytosolic enzyme specialized for the rate-determining step of *de novo* lipogenesis, while ACC2 is a mitochondrial membrane-related enzyme. The malonyl-CoA produced, is mainly involved in the allosteric inhibition of carnitine palmityl transferase (CPT1) and participates in the transport of long-chain fatty acids (LCFAs) to the mitochondria [35]. Studies have shown that the knockout of ACC1 reduces malonyl-CoA levels and affects malonylation; this suggests a direct relationship between malonyl-CoA, ACC1-catalyzed malonyl-CoA production and malonylation [24]. In the heart, malonyl-CoA is synthesized by acetyl-CoA carboxylase and degraded by malonyl-CoA decarboxylase (MCD). Furthermore, it acts as a potent endogenous inhibitor of cardiac fatty acid oxidation. Additionally, lysine malonyl-CoA could participate in cell regulation of energy homeostasis in response to the dynamic intracellular concentration of malonyl-CoA [36].

MCD catalyzes the conversion to malonyl-CoA to carbon dioxide and acetyl-CoA. It facilitates the maintenance of metabolites levels and regulates the activity of peroxisomes and mitochondria [37]. With increased malonyl-CoA levels in MCD-deficient cells, malonylation of substrate proteins increases. In addition, MCD reduces the allosteric inhibition of CPT1 by malonyl-CoA, which is an important rate-limiting step in mitochondrial LCFA β-oxidation, thereby enhancing the process [38].

## 3. Malonylation and related diseases

### 3.1 Metabolic diseases

Metabolic pathways, such as glutamine metabolism, fatty acid oxidation, and glycolysis, play unique roles in angiogenesis [39]. EC metabolism has only recently been recognized as a driver of angiogenesis and is severely disrupted in cancer and diabetes. As tumor ECs show increased glycolysis, reducing this metabolic activity promoted treatment efficacy in preclinical tumor models [40] (Table 2).

**Table 2:** The critical contribution of malonylation to various disease and plant.

Disease	Enzyme	Cell	Pathway	Function	Reference
Vascular defects	Malonyl-CoA	Endothelial cells	mTOR	Protein synthesis, Cell size, Proliferation	[58]
Malonic aciduria	Malonyl-CoA Decarboxylase, Malonyl-CoA	Nephrocyte	/	Regulate β-oxidation	[59, 71]
UC	Malonyl-CoA	Macrophage	MAPK	Inflammation, TNFα translation	[24, 70]
Diabetes	Malonyl-CoA, Malonyl-CoA Decarboxylase	Liver cell	Diagram of glucose and fatty acid metabolic	Regulation of enzymatic activity of glucose metabolism	[19]
Cardiovascular disease	Malonyl-CoA, Malonyl-CoA Decarboxylase	Cardiomyocyte	/	Enhancing cardiac function, Preventing ischemic injury	[80, 83]
Neurofibromatosis type 2	Malonyl-CoA, CPT1C	Somatocyte	mTOR	Lipid metabolism	[88]

### 3.1.1 Diabetes

Type 2 diabetes is characterized by a relative resistance to peripheral insulin action, resulting in impaired glycemic control [41], production of glycated proteins and hyperglycemia. Elevated levels of malonyl-CoA have been reported in prediabetic rats and patients with type 2 diabetes [42, 43]. In addition, overexpression of MCD in hepatocytes reverses insulin resistance and reduces malonyl-CoA levels [44]. As a malonyl donor in lysine malonylation, malonyl-CoA may influence diabetes and obesity [45]. Using proteomic and affinity enrichment experiments, Du et al. [19] discovered 573 malonylated lysine sites in 268 proteins, in the liver tissues of WT and db/db mice. Based on bioinformatic analysis, these proteins were found to regulate fatty acid and glucose metabolism. Interestingly, they also found liver-specific elevation in the levels of malonylated lysine and proteins in the liver tissues of db/db and ob/ob mice (both being typical models of type 2 diabetes [46, 47]) compared to those in wild-type (WT) mice; five proteins with increased lysine malonylation levels, namely glucose-6-phosphate isomerase (G6PI), 10-formyl-tetrahydrofolate dehydrogenase (10-FTHFDH), lactate dehydrogenase A (LDHA), fructose bisphos-phatase 1 (FBP1), and aldolase B (ALDOB), were further validated using western blot and immunoprecipitation analyses. In certain other animal models, including type 2 diabetic monkeys, ob/ob rats, and KK mice (a model of glucose intolerance and insulin resistance [48]), different degrees of elevation of lysine malonylation have been reported. Taken together, these findings suggest that the liver-specific elevation of lysine malonylation may be common in type 2 diabetes. Furthermore, another recent study [49] reported that hepatic overexpression of SIRT5 ameliorated metabolic abnormalities in ob/ob mice, possibly through de-malonylated proteins in a major metabolic pathway. Consequently, SIRT5 and malonylation may be potential targets for type 2 diabetes treatment.

Altogether, these findings imply a latent regulatory function for protein malonylation in type 2 diabetes, which could inform the development of novel treatment strategies.

### 3.1.2 Diabetic retinopathy

Diabetic retinopathy (DR), a retinal microangiopathy [50], is a specific microvascular complication of diabetes mellitus and is fairly common. It is the major cause of vision loss in the elderly. Hyperglycemia and altered metabolic pathways lead to oxidative stress and neurodegeneration in the early stages of DR. Endothelial damage, development of microaneurysms and punctate intraretinal hemorrhage are early signs of non-proliferative DR [51]. Abnormal lipid metabolism is known to be a risk factor for microvascular dysfunction, a main cause of visual impairment in DR [52]. A recent study found that double hydrogen artemisinin (DHA), an active metabolite of artemisinin, reduces fatty acid synthase (FASN) expression, thereby increasing malonyl-CoA levels in the cell. Furthermore, FASN, a key enzyme in lipid metabolism has been shown to be elevated in DR. It drives palmitate synthesis with malonyl-CoA as the substrate. This enhances the malonylation of the mechanistic target of rapamycin (mTOR) at lysine 1218 (K1218), resulting in the suppression of mTOR complex 1 (mTORC1) activity. With this signaling cascade, DHA inhibits cell proliferation and tube formation in human retinal microvascular ECs (HRMECs) and protects against vascular injury in diabetic mice. DHA has been known to show such anti-angiogenic activity in a variety of diseases [53]. A previous study [54], elucidated the potential roles of FASN and lysine malonylation in vascular injury in DR and described the importance of DHA as a therapeutic agent for lysine malonylation.

### 3.1.3 Vascular disorder

**Figure 2.**
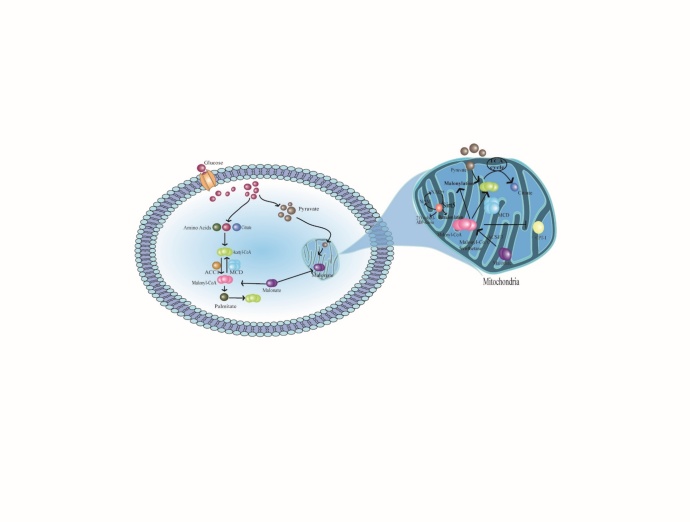
Malonylation mediated signaling pathways in endothelial cells and macrophages. Malonylation of mTOR in endothelial cells inactivates it and leads to defective angiogenesis. Upon activation of macrophages with LPS, accumulation of citrate can be converted to malonyl-CoA in an ACC1-dependent manner. Malonyl-CoA can then contribute to an increase in lysine malonylation. One of the substrates for this modification is GAPDH which could release TNF-α-bound RNA after undergoing malonylation, making it available for translation. FASN, Fatty Acid Synthase; Acetyl-CoA, Acetyl-Coenzyme A; Malonyl-CoA, Malonyl Coenzyme A; mTOR, mechanistic target of rapamycin; mTOR C1, mTOR complex 1; LPS, Lipopolysaccharide; TLR, toll-like receptors; GAPDH, glyceraldehyde-3-phosphate dehydrogenase; TNF-α, tumor necrosis factor alpha; IL-1β, Interleukin-1 beta; IL-6, Interleukin 6.

Vascular disorder is broadly defined as the presence of lesions on blood vessels throughout the body, including arteries, veins, and capillaries [55, 56]. In clinical practice, the diseases presenting with arterial lesions include atherosclerosis, abdominal aortic aneurysm, aortic aneurysm, thoracic aortic aneurysm, and inflammatory arterial vascular lesions. In this section, we focus on the relationship between malonylation and angiogenesis. Angiogenesis is dependent on pro- and anti-angiogenic molecules that regulate endothelial cell proliferation and migration. Well-regulated angiogenesis plays a key role in many physiological conditions such as reproduction and embryonic development [57]. FASN mediates *de novo* lipid synthesis by catalyzing the conversion of acetyl-CoA and malonyl-CoA to palmitic acid, using nicotinamide-adenine dinucleotide phosphate (NADPH) [58] (Fig. 2).

In a study by Bruning et al. [58], loss of FASN was found to inhibit angiogenesis in endothelial cells. FASN knockdown (FASN^KD^) inhibited EC proliferation and vascular sprouting by increasing malonyl-CoA levels. Moreover, FASN blockers have reduced pathological ocular neovascularization at doses 10-fold lower than that used in anticancer therapy. Thus, the regulation of EC function by FASN is mediated through malonyl-CoA. By performing a proteomic screening with an anti-malonylation antibody and a malonylation peptide, the authors have demonstrated that elevated levels of malonyl-CoA affected protein malonylation, similar to the findings of Colak et al. [59]. They also found that mTOR, a threonine/serine kinase activated by anabolic signaling, showed increased levels of malonylation in orlistat-treated or FASN^KD^ ECs. Blocking FASN elevated mTOR activity and promoted malonylation, thereby leading to angiogenesis and vascular defects. Similar results have been observed for malonylation of mTOR using malonyl-N-acetylcysteine (NaC), a cellular penetrant that can directly induce lysine malonylation [60] independent of FASN inhibition; inhibition of mTORC1 activity during FASN silencing or blocking was not an indirect action but a direct effect of the malonylation of mTOR. These results suggest that angiogenic disorders are not caused by redox imbalance, energy stress, or palmitate depletion, but by the resultant increase in malonyl-CoA. The authors found that mTOR K1218 malonylation had a profound effect on vascular generation. FASN in ECs indirectly controls the PTM of target proteins, especially mTOR, which is a primary metabolism regulator, through lysine malonylation. Malonylation of mTOR at K1218 following FASN inhibition reduces the enzymatic activity of mTORC1, impairs its kinase activity, and reduces the phosphorylation of downstream targets, such as 4EBP1/p70S6K [54]. Silencing of ACC1 normalizes malonyl-CoA levels and reactivates mTOR in FASN^KD^ ECs. Mutagenesis has revealed a key role of mTOR malonylation in angiogenesis. These findings have revealed a new role of FASN in metabolite signaling and have elucidated the antiangiogenic effect of FASN blocking via malonylation.

### 3.1.4 Osteoarthritis (OA)

OA is the most commonly diagnosed disease of the musculoskeletal system and manifests clinically as a true inflammatory feature of synovitis. It is presumed to include signs of inflammation that are sometimes histologically indistinguishable from rheumatoid synovial infiltrates [61]. OA is one of the most common forms of arthritis and the leading cause of disability worldwide. Since the mid-20th century, the prevalence of OA in the U.S. has doubled [62]. Obesity and other metabolic disorders are potential risk factors for the development of OA [63]. In addition to increased joint load, systemic metabolic factors may play a prominent role in the development of obesity-related OA. Systemic metabolic disorders disrupt various cellular processes, such as glycolysis, mitochondrial bioenergy, and nutrient sensing. Under abnormal metabolic conditions, such as type 2 diabetes, malonylation can occur throughout metabolically active tissues, such as the liver [19], and lead to metabolic dysfunction. Increased protein malonylation in chondrocytes has been associated with metabolic dysregulation and the occurrence of OA. High levels of protein malonylation have been found in the liver of db/db mice. Studies have consistently found higher levels of malonylation in the cartilage of obese mice fed a high-fat diet or with obesity induced by pro-opiomelanocortin (POMC) deficiency. Furthermore, SIRT5 deficiency in the cartilage resulted in the upregulation of malonylation, which in turn caused an early OA-like phenotype [64].

### 3.2 Inflammation

Lysine malonylation has been identified as a novel mechanism by which GAPDH, a glycolytic enzyme, regulates the production of proinflammatory cytokines [24]. The administration of GAPDH exerts an anti-inflammatory effect by reducing tumor necrosis factor alpha (TNFα) levels in a mouse model of sepsis [65]. Luke et al. [24] found that GAPDH exerted an inflammatory effect by promoting the translation of TNF-α through malonylation in a mouse model of sepsis. First, the authors identified the role of malonyl-CoA in cytokine production in macrophages, and then demonstrated that the activation of macrophages leads to protein malonylation. Elevated levels of protein malonylation induced by the gram-negative bacterial product lipopolysaccharide (LPS) has been shown to result from the accumulation of malonyl-CoA. Furthermore, it was confirmed that K213 of GAPDH was especially sensitive to malonylation and responded to an increase in cytoplasmic malonyl-CoA levels following LPS treatment [66]. Normally, GAPDH binds to and represses the translation of several inflammation-associated mRNAs, including that encoding TNFα. Following malonylation, GAPDH segregates from the TNFα mRNA and promotes translation. Hirschey and Zhao [66] further found that GAPDH malonylation at K213 was involved in the regulation of gene translation, particularly TNFα. Studies have found that malonylation is not only associated with angiogenesis in ECs but also acts as an inflammatory signal in macrophages (Fig. 2).

Further characterization of malonylation is required to understand its role in inflammation and infection and identify effective therapeutic agents.

Ulcerative colitis (UC), a chronic inflammatory bowel disease [67] of unknown origin, is characterized by mucositis in the rectum that gradually extends to the proximal colon. The typical manifestations of UC include rectal urgency, bloody diarrhea, rush, and different degrees of abdominal pain that can be alleviated by defecation [68]. UC is a risk factor for colon cancer and affects the quality of life of patients. Advances in medical treatment have led to a paradigm shift in treatment goals from symptom relief to endoscopic and histological healing to achieve better long-term outcomes. As a result, diagnostic methods have improved to monitor disease progression. Despite these improvements in patient care, a significant proportion of patients, who do not respond to drug therapy or develop colitis-related colorectal dysplasia or cancer, still require rectocolectomy [67]. Natural compounds isolated from fruits, herbs, and vegetables have been reported to be effective in treating mice with dextran sodium sulfate-induced UC. Intraperitoneal injection of atractylodin, a bioactive component of *Atractylodes*, inhibited the progression of rheumatoid arthritis in mice, as evidenced by reduction in clinical arthritis scores, paw swelling, and histopathological changes in the joints [69]. Liu et al. [70] first demonstrated that atractylodin can improve the pathological symptoms and restore the intestinal barrier of UC mice. The authors found that TNF-α was highly expressed in the lysates and supernatants of LPS-treated macrophages; however, after treatment with various atractylodin concentrations, its levels decreased to varying degrees. Atractylodin and GAPDH showed binding-free energy of 5.42 kcal/mol in molecular docking experiments, suggesting that GAPDH may be an anti-inflammatory target. GAPDH activity was found to decrease gradually with increasing atractylodin concentration. However, GAPDH protein expression was unaffected. Western blot analysis confirmed that LPS treatment (100 ng/mL) for 24 h markedly enhanced protein malonylation in macrophage lysates, and an immunoprecipitation assay showed that atractylodin noticeably inhibited the malonylation of the GAPDH protein.

### 3.3 Malonic aciduria

Malonic aciduria [71] is an extremely rare inherited metabolic disease caused by MCD deficiency and is characterized by systemic clinical involvement, digestive and neurological symptoms, hypoglycemia, metabolic acidosis, developmental delay, cardiomyopathy, and seizures [72, 73]. The protein substrate for SIRT5-mediated lysine malonylation remains unknown [59]. Proteomic analysis identified 4,943 malonylation sites on 1,822 proteins and 4,042 malonylation sites on 1,426 proteins in human fibroblasts and mouse liver tissues, respectively. Increase in the number of MCD-deficient cells is known to lead to an increase in malonic acid and malonyl-CoA levels and induce malonylation [59]. In MCD-deficient cells, 461 malonylation sites were identified with a >2-fold increase in response to MCD deficiency. In MCD^-/-^ fibroblasts, and 1,452 malonylation sites were detected, unlike in MCD^+/+^ cells, indicating that malonylation caused by MCD deficiency has a pathogenic role.

Colak et al. performed proteomic screening, affinity enrichment, and HPLC-MS/MS analysis of malonate substrates in MCD-deficient fibroblasts from patients with malonic aciduria and the livers of SIRT5 knockout (KO) mice. Compared with those in the WT control, MCD-deficient cells showed altered levels of the critical malonylated substrate in malonic aciduria; cells with high lysine malonylation rates had impaired fatty acid oxidation and mitochondrial function. These findings emphasize the role of lysine malonylation in the pathophysiology of malonic aciduria [59].

A recent study [24] has elucidated the mechanisms of how macrophages regulate the expression of inflammation-related factors through lysine malonylation of proteins *in vivo*. The study also demonstrated how proteins exert regulatory functions through malonylation that influence protein conformation and interactions with other molecules.

As techniques to identify malonic aciduria have matured, the study by Colak et al. [59], linking malonylation to malonic aciduria has been a major breakthrough. However, the underlying mechanisms and the targets of malonylation in this disease remain unclear and requires future investigation.

### 3.4 Cardiovascular disease (CVD)

CVD is the leading cause of morbidity and mortality worldwide [74, 75], and metabolic dysfunction is the core mechanism underlying CVD. Previous studies of CVD have focused on the role of LCFAs. However, an increasing number of studies have demonstrated that short-chain fatty acids, including propionic, malonic, butyric, 2-hydroxyisobutyric (2-HIBA), β-hydro-xybutyric, crotonylation, succinic, and glutaric acids, and their homologous acylation are involved in CVD [76, 77].

Mitochondrial reactive oxygen species (ROS) contribute to endothelial dysfunction and hypertension [78, 79]. Malonate attenuates mitochondrial ROS production in angiotensin II-induced hypertension. Inhibition of MCD [80-82] leads to increased levels of malonyl-CoA, an intracellular inhibitor of mitochondrial FA uptake. This in turn reduces FA oxidation and enhances glucose oxidation, ultimately leading to reduction in toxic by-products, thereby enhancing cardiac function and cardiac efficiency and preventing myocardial ischemic injury. Furthermore, malonate is a potent inhibitor of mitochondrial succinate dehydrogenase (SDH), which aggravates mitochondrial ROS production [83]. For instance, through the regulation mitochondrial ROS levels, intracoronary malonate inhibition of SDH in pigs lead to a significant reduction in the myocardial infarct size. This prevented the infarction from progressing to transient coronary occlusion and resulted in a partially improved functional recovery [5].

To date, malonylation-related enzymes have been shown to be closely associated with cardiac diseases, as well as with ROS. However, research on this aspect has stagnated. An in-depth study of the involved mechanisms and the targeted regulation of malonylation can provide insights into biomolecular targets for the treatment of cardiomyopathy.

### 3.5 Tumorigenesis

Kulkarni et al. developed a chemical proteomics approach that segregates thioester reaction from enzyme utilization [60], enabling the selective enrichment of non-enzymatically acylated targets. Many such [84] targets were identified, including several enzymes with low glycolytic activity.

According to functional studies, malonyl-CoA is an active thioester metabolite that can alter and inhibit glycolytic enzyme activity. These studies focused on glycolytic enzymes and evaluated GAPDH and the M2 isoform of pyruvate kinase 2 (PKM2) as prototypical targets for thioester reactivity and investigated the ability of acyl-CoA metabolites to inhibit the activity of purified GAPDH [85-87]. While most acyl-CoAs catalyzed GAPDH, pre-incubation of GAPDH with malonyl-CoA resulted in potent inhibition of enzymatic activity. Additionally, malonyl-CoA was found to inhibit PKM activity in the human lung cancer cell line, A549 [60].

Kulkarni et al. examined the scope and the mechanistic basis of the effect of malonyl-CoA on the glycolytic enzyme functions. Besides the covalent inhibitory mechanism, GAPDH activity was inhibited by malonyl-CoA in a time-dependent manner. The reactivity of malonyl-CoA was considerable, and an increase in malonyl-CoA reactivity may be related to its carboxyl functional group, which induces electron absorption, thus increasing the sensitivity of adjacent thioesters to nucleophilic attack. UV spectroscopic analysis generated consistent results; the simplified malonyl thioester cleaved more rapidly than its acetyl analog. Overall, these findings demonstrated the sensitivity of GAPDH to non-enzymatic acylation inhibition and emphasized the importance of malonyl-CoA as a reactive cytosolic metabolite. Having demonstrated *in vitro* that cytosolic malonyl-CoA inhibits glycolytic enzyme activity through covalent malonylation, the authors subsequently determined that manipulation of malonylation in cells could similarly affect enzymatic activity.

Neurofibromatosis type 2 (NF2) is usually caused by an inactivating mutation and is an autosomal dominant cancer susceptibility syndrome [88]. NF2 is characterized by multiple low-grade tumors along the central and peripheral nervous systems and is associated with disease manifestations, such as meningiomas, benign schwan-nomas (including bilateral vestibular schwannomas), and ependymomas. Although these lesions are benign, NF2 is usually fatal because it involves the development of intracranial tumor, which is inoperable. In addition, somatic mutations in NF2 can cause malignant mesothelioma, among other complications. NF2-deficient cells showed an increased dependence on lipid synthesis[88]. FASN inhibitors have been effective in the treatment of NF2-related disorders, either alone or in combination with other drugs, that increase malonyl-CoA levels. In fact, changes in lipid metabolism of NF2-mutated cells after FASN blockade are related to increased levels of malonyl-CoA.

This demonstrates a novel targeting strategy for NF2-deficient tumors and indicates that enzymes participating in lipid metabolism may be potential therapeutic modalities for cancer. Since lysine malonylation is closely related to fatty acid metabolism, clarifying the mechanism of malonylation in NF2 could further facilitate targeting NF2 to reduce its morbidity and mortality.

The fact that malonyl-CoA can be bound by carnitine palmitoyltransferase 1C (CPT1C) [89] suggests that CPT1C is a sensor of neuronal lipid metabolism that appears to influence triacylglycerol (TAG) and ceramide metabolism. CPT1C KO mice exhibited various brain disorders, including impaired spatial and cognitive learning, abnormal food intake, disturbed energy balance, and motor disorders. The first pathogenic variant of CPT1C has recently been reported in humans, and *CPT1C* has been identified as the gene responsible for hereditary spastic paraplegia [90]. Therefore, CPT1C, through the malonyl-CoA pathway, is a potential target for cancer treatment.

Although studies investigating the role of malonyl-CoA in clinical contexts are lacking, malonylation is likely to be a key target for tumor treatment, given the definitive results of animal experiments.

## 4. Others: lens protein

The lens protein conversion rate is negligible, and therefore accumulates PTM throughout the aging process. PTMs such as carbamylation, phosphorylation, methy-lation, deamidation, advanced glycosylation, and oxidative modification, have been identified in human lens proteins in aging and cataracts [91-93].

Acylated lysine residues are the main chemical modifications in lens proteins [94]. Investigation of the proteins undergoing acylation in aging human lens revealed multiple proteins with modified propionylated (PropK) and malonylated (MalK) lysine residues. In the water-soluble (WS) and water-insoluble solubilized (WIS) protein fractions, malonylation and propionylation were observed primarily in a protein above the 20-kda marker [94] by western blotting, indicating that the immunoreactivity of the αB-crystallin increased with sample age. It is noteworthy that this study was the first report on the malonylation of human lens proteins.

In cultured human lens epithelial cells, both SIRT3 and SIRT5 are catalytically active and they depropionylate and demalonylate acylated lysozymes. However, no deacylase activity of SIRT3 and SIRT5 has been observed within the human lens WS protein fraction, suggesting that these proteins are active in lens epithelial cells, but not in fibrocytes [95]. This finding may be due to the active metabolism of lens epithelial cells, which undergo differentiation and proliferation. The regulation of propionylation and malonylation may play a role in these processes. Further research on lens epithelial cells is needed to understand the significance of acylation and its regulation [94].

## 5. Clinical perspectives

Since its discovery, malonylation has not yet found clinical applications as a therapeutic target. Given below is a discussion on the reports of enzymes that are closely related to malonyl-CoAs, and specifically, their use in clinical settings.

MCD or malonate deficiency is an inborn metabolic disorder caused by mutations in MCD that reduce or eliminate enzyme activity and inhibit the conversion of malonyl-CoA to acetyl-CoA [96]. Multiple medical conditions are associated with congenital MCD deficiency, some of which are also common in fatty acid oxidation disorders, including muscle weakness, hypoglycemia, and cardiomyopathy [81, 97, 98]. Patients with an MCD deficiency may also experience delayed neurodevelopment. Although the etiology of this effect is unknown, it may be due to disruption of the interaction between CPT1 and malonyl-CoA. This finding implies that inhibition of CPT1 by the addition of malonyl-CoA may play a prominent role in the pathophysiology of MCD deficiency [99]. In fact, oxidation of palmitic and myristic acids was severely reduced in fibroblasts from patients with MCD deficiency, suggesting that malonyl-CoA might be critically involved in the pathogenesis of this disease by inhibiting fatty acid oxidation. According to these results, the accumulation of malonyl-CoA could influence metabolic pathways through CPT1-independent malonylation. Increase in mitochondrial lysine malonylation in MCD deficiency plays a pathogenic role. Patients with MCD deficiency had high blood levels of malonylcarnitine and high levels of organic acids, such as malonate, in their urine [100]. Inhibition of fatty acid catabolism caused by high levels of malonyl-CoA appears to contribute, at least partly, to the onset of the disease. Researchers have recently found elevated malonylation levels in cells from MCD^-/-^ patients [101]. Therefore, malonylation may be a significant mechanism that mediates the pathophysiology of MCD deficiency. The elevated malonylation of several mitochondrial proteins may represent another pathological mechanism associated with malonic aciduria, which could be a rational diagnostic biomarker and therapeutic target for MCD deficiency [59].

Preclinical studies suggest that inhibition of MCD may ameliorate the progression of heart failure, ischemia, and insulin resistance [80, 102-106], and improve heart function in patients with cardiac disease. As an endogenous inhibitor of fatty acid oxidation, malonyl-CoA influences cardiac energy metabolism and function; studies have shown that inhibition of malonyl-CoA by MCD increased fatty acid oxidation [102, 103, 107-109]. In a previous study, increased cellular malonyl-CoA levels led to reduced uptake of LCFAs into the mitochondria, resulting in reduced fatty acid oxidation rates. Taken together, these findings suggest that the inhibition of fatty acid oxidation can improve the efficiency and functioning of the heart. Therefore, clinical regulation of malonyl-CoA levels is a potential strategy for treating several types of heart diseases, such as heart failure and ischemia/reperfusion, as well as insulin resistance [110]. The levels of malonyl-CoA are regulated by two proteins in the heart: MCD and ACC. MCD decarboxylates malonyl-CoA to acetyl-CoA, while ACC synthesizes malonyl-CoA from acetyl-CoA. Thus, there are two major approaches to increase cardiac malonyl-CoA levels: decreasing MCD activity or increasing ACC activity [111].

Manipulation of malonyl-CoA metabolism as a part of chemotherapeutic strategies has been receiving increasing attention. For instance, the *in vitro* inhibition of MCD establishes toxic conditions for breast cancer cells [112]. Using metabolic modeling strategies, MCD inhibition has been identified as a critical metabolic vulnerability in several cancer cell lines [113]. Furthermore, SIRT5-catalyzed removal of GAPDH malonylation enhances its enzymatic activity [18], while SIRT5 KO reduces lung cancer cell growth *in vitro* and *in vivo* [114]. Recent studies have shown that the anticancer effects of altered malonyl-CoA metabolism may be partially mediated by functional changes caused by protein malonylation.

## 6. Discussion

PTMs play an important role in the pathogenesis of several diseases. Recent developments in proteomics have led to the discovery of diverse novel lysine-acyl modifications and established the biological relevance of protein acylation.

An increasingly diverse group of PTM proteins is involved in the reversible acylation of lysine. The abundance, structural diversity, and dynamics of acylproteomes suggest that their roles in the regulation of cellular functional networks are complex. Therefore, elucidating the dynamic processes and outcomes of the “acylation code” is important to understand its impact on health and disease. To achieve this goal, researchers have analyzed malonylated markers using various methods. Recent studies have found significant differences among malonylation, succinylation, and acetylation in their distribution, target proteins, and modified individual lysine residues. Malonyl-CoA is a donor and can influence the dynamics of malonylation. However, most studies only describe malonyl-CoA activity without considering the level of modification.

Several experiments have identified malonylation substrates in the nucleus and the cytoplasm of human fibroblasts, suggesting the presence of a malonyl transferase. This process has been proposed to occur in a non-enzymatic manner in the high pH chemical environment of the mitochondria. However, spontaneous *in vitro* protein acylation does not rule out the possibility of enzyme catalyzed PTM reactions. Considering that the cytoplasmic and nuclear pH is lower than the mitochondrial pH, and the intracellular localization of the malonylation substrate in the liver is different from that of fibroblasts, the reactions in the extramitochondrial medium of human fibroblasts are particularly enzymatic. Malonylation plays different roles based on the involved signaling pathways in different disease conditions. Functional studies have emphasized that malonyl-CoA, can alter the activity of glycolytic enzymes, as a reactive thioester metabolite. Synthetic thioesters can be used as reagents for non-enzymatic acylation of living cells. Multiple studies [60, 115] provide novel insights into the targets and mechanisms of non-enzymatic acylation and demonstrate the utility of reactivity-based approaches for experimental analysis of this phenomenon in biology and diseases.

Several immediately relevant research questions regarding protein acylation remain to be unanswered. For instance, it would be interesting to determine the molecular triggers and regulators of protein malonylation and demalonylation. Regardless of the mechanism, validation of malonylation targets is essential in assessing the effects of protein malonylation on cell physiology. Identifying the target will not only provide a better understanding of the mechanism of malonylation but will also provide greater possibilities for its clinical applications. Targeted therapies for disease and known targets for malonylation do not often coincide. Therefore, additional techniques are required to identify new targets and link them to disease treatment, which is often difficult and time-consuming. Several studies have indicated that protein function is not the only factor influenced by lysine malonylation, and perhaps particular attention needs to be paid to the effects of malonylation on protein-protein interactions, structure, cellular localization and relevant parameters. Many studies focused on malonylation-related enzymes and investigated whether the associated diseases are directly linked to malonylation in both normal and disease tissues. It is likely that future research will continue in this direction, identify more malonylation-associated diseases, and develop powerful analytical tools that provide robust results. Research on malonylation will inevitably move towards the clinic, as well as provide treatment strategies for diseases. The role played by PTMs is likely to be different due to the manifestation of disease symptoms in different species and their role in the regulation of immunometabolic gene expression. This also poses a great challenge for the study of malonylation. Sample collection is difficult, and the involved ethical issues require considerable attention from researchers.

Recent studies on histone lysine malonylation have reported a novel PTM of proteins that reveals the complexity of malonylation [116]. Malonylation may be primarily regulated by malonyl-CoA metabolism, and different conditions alter malonylation levels. The relationship between disease and histone malonylation has been identified, suggesting that histone malonylation could be a future therapeutic target. Pharmacologically targeting regulatory enzymes would require the development of specific modulators (activators and inhibitors) and could be potential therapeutic approaches for cancer and metabolic diseases. Exploring effective methods for identifying malonylation sites can help in the study of disease treatment and related drugs.

Particular attention should be paid to protein identification to accelerate research progress. Unfortunately, the available bioinformatic tools are ineffective in distinguishing modified proteins from small molecules. The big question here pertains to the conditions required for the growth of cells used for these analyses. Ideally, one should establish conditions that generate phenotypes that can be biochemically and genetically analyzed. Nevertheless, the generation of novel experimental tools offers new opportunities to better understand metabolite-protein interactions. However, there are some important issues associated with these tools. It must be verified if mitochondrial proteins can be assessed using highly acylated probes. Furthermore, it must be investigated if individual chemical proteomic probes are unique to a particular acyl-CoA species or if require a thioester-reactive universal probe is required. Following this, the question remains if the probe-protein reactivity reflects the reactivity of acyl-CoA that the probe mimics, or the possibility of the lysine of a particular protein being modified. Substantial research work is needed to fully understand the prospects and the functional consequences of protein acylation and the balance between enzymatic and non-enzymatic acylation. Recent evidence has also suggested that the associated histone modifications affect gene expression [23]. Thus, future studies should also investigate malonylation and the metabolic regulation of gene expression.

## 8. Conclusions

Over the past decade, important advances have been made in unraveling the scope and mechanisms of malonylation and its cellular function. Recent advances in high-resolution quantitative mass spectrometry, selective chemical probe development, and genome engineering have provided improved opportunities for detailed mechanistic studies and system-wide investigations to address future challenges.
